# Impaired dermal wound healing in discoidin domain receptor 2-deficient mice associated with defective extracellular matrix remodeling

**DOI:** 10.1186/1755-1536-4-5

**Published:** 2011-02-02

**Authors:** Elvira Olaso, Hsin-Chieh Lin, Li-Hsien Wang, Scott L Friedman

**Affiliations:** 1 Department of Cell Biology and Histology, University of the Basque Country School of Medicine, Leioa, Spain; 2 Regeneron Pharmaceuticals, Incorporated, Tarrytown, NY, USA; 3 Division of Liver Disease, Department of Medicine, Mount Sinai School of Medicine, New York, NY, USA

## Abstract

**Background:**

The wounding response relies on tightly regulated crosstalk between recruited fibroblasts and the collagenous extracellular matrix (ECM). Discoidin domain receptor 2 (DDR2) is a tyrosine kinase receptor for fibrillar collagen expressed during pathologic scarring, for example wound healing, arthritis and cancer. We have previously shown that DDR2 phosphorylation drives key wounding responses in skin fibroblasts including proliferation, chemotactic migration and secretion of both metalloproteinases and fibrillar collagen. In this study we compared healing of cutaneous wounds in DDR2^+/+ ^and DDR2^-/- ^mice and analyzed specific fibroblast responses.

**Results:**

Cutaneous wound healing was significantly delayed in DDR2^-/- ^mice compared with DDR2^+/+ ^animals. Reduced α-smooth muscle actin (αSMA) expression and matrix metalloproteinase 2 (MMP2) activity in the DDR2^-/- ^wound extracts indicated defective recruitment of skin fibroblasts. DDR2^-/- ^wounds showed decreased tensile strength during healing, which correlated with a significant reduction in collagen content and defective collagen crosslinking. Non-wounded skin in DDR2^-/- ^mice expressed less mRNA of the crosslinking enzymes lysyl oxidase (LOX), lysyl hydroxylase1 (LH1) and matricellular 'secreted protein, acidic and rich in cysteine' (SPARC; also known as osteonectin). Skin fibroblasts isolated from DDR2^-/- ^mice displayed altered mRNA expression of a cluster of collagens, proteoglycans, integrins and MMPs that have been previously correlated with DDR2 expression, and reduced LOX, LH1 and SPARC mRNA levels and proteins. Stable reconstitution of wild-type DDR2 by retroviral infection restored LOX, LH1 and SPARC mRNA and protein levels in DDR2^-/- ^fibroblasts. Contraction of collagen gels was reduced in DDR2^-/- ^fibroblasts, accompanied by significantly reduced phosphorylated Src^Y418^. Inhibition of either LOX activity by β-aminoproprionitrile or MMP activity by *N*-[(2R)-2-(hydroxamido carbonylmethyl)-4-methylpentanoyl]-l-tryptophan methylamide (GM6001) reduced collagen gel contraction by skin fibroblasts after DDR2 induction with soluble collagen type I.

**Conclusions:**

DDR2 contributes to skin fibroblast responses during tissue injury. Defective synthesis of collagen type I, crosslinking molecules and MMP2 predispose DDR2^-/- ^mice to defective dermal wounding.

## Background

Collagen receptors mediate fibroblast responses during tissue regeneration and healing. Integrins are the most studied collagen receptors (for review, see [1]). Integrins signaling results in fibroblasts recruitment to the wounded area and synthesis of extracellular matrix (ECM) components to conform the healing tissue. Integrins also function as mechanotransducers of the tensile strength exerted by the healing matrix [2,3] to further activate downstream signaling that drives fibroblast contraction of the wound [4-6]. In addition to integrins, the discoidin domain receptor (DDR) family of receptors (DDR1 and DDR2) also interact with the collagenous ECM. As opposed to integrins, DDRs are tyrosine kinase receptors, and thus become phosphorylated in response to collagen [7]. The molecular details of collagen recognition by DDRs are starting to become known [8]. DDR1 preferentially binds to collagen type I. DDR2 has higher specificity for fibrillar type I collagen than DDR1, but also for collagen type II through a specific DDR2 recognition site in the D2 period of collagen II [9]. DDR overexpression is associated with fibrotic diseases of the lung, kidney and liver [10], atherosclerosis, osteoarthritis [11], as well as several tumors of epithelial origin [12,13]. DDR2 mRNA is also upregulated in dermal burn wounds [14]. Mice deficient in DDR2 present a dwarfed phenotype with reduced proliferative response of experimentally wounded skin compared to wild-type littermates [15].

Collagen receptor signaling results in matrix metalloproteinase (MMP) release [16], and dysregulation of MMP activity is a key feature of defective wounding response. MMP2 is the predominant protease in dermal wound healing [17] and MMP2 activity is reduced in DDR2^-/- ^skin and cultured DDR2^-/- ^skin fibroblasts, with lower MMP2-dependent cell proliferation and chemotactic invasion [18].

The role of collagen receptor signaling in response to tissue stiffness is becoming an area of intensive research [4,19]. Stiffness of the healing tissue depends on fibrillar collagen formation and its covalent crosslinking. Deficient crosslinking machinery results in hyperelastic tissue that scars easily, and heals slowly and poorly [20], whereas excessive collagen crosslinking is a hallmark of tissue fibrosis [21]. Fibroblasts synthesize ECM components such as the matricellular glycoprotein 'secreted protein, acidic and rich in cysteine' (SPARC; also known as osteonectin) that modulates collagen fibril growth and deposition [22], and synthesize two of the enzymes that mainly mediate collagen crosslinking: lysyl oxidase (LOX) and lysyl hydroxylases (LH) [23].

In the present study, we investigated the ability of DDR2^-/- ^mouse skin to heal. The skin wounds of DDR2^-/- ^mice heal more slowly than DDR2^+/+ ^mice, and display diminished tensile strength, reduced numbers of recruited α smooth muscle actin (αSMA)-expressing fibroblasts and decreased MMP2 and collagen levels, with reduced crosslinking and modified mRNA expression of several proteoglycans, collagens, integrins and MMPs. These findings are explained by reduced expression of the crosslinking molecules LOX, LH1 and SPARC in dermal DDR2^-/- ^wounds and altered Src-dependent fibroblast contractile ability. The data implicate DDR2 in modulation of collagen processing and tensile wound strength during tissue healing.

## Results

**Figure 1:**
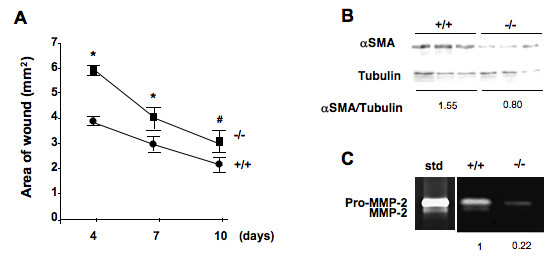
**Impaired experimental skin wound healing in discoidin domain receptor 2 (DDR2)^-/-^ mice correlates with defective fibroblast recruitment and MMP2 activity**. (a) Impaired experimental skin wound healing in discoidin domain receptor 2 (DDR2)^-/- ^mice. Following experimental wounding, the area of the remaining wound was measured using a caliper (n = 12). *P < 0.001, and #*P *< 0.05, significantly different compared with DDR2^+/+ ^group. **(b) **Representative western blot analysis of α smooth muscle actin (αSMA) expression in skin tissue extracts from three DDR2^-/- ^and three DDR2^+/+ ^mice, wounded 7 days before. Tubulin expression was used as control for protein loading. Bands were semiquantified by scanning densitometry and results expressed as a ratio αSMA/tubulin to reflect average density of fibroblast per wound. **(c) **Representative gelatin zymography in DDR2^-/- ^and DDR2^+/+ ^skin tissue extracts wounded 7 days before. Bands from pro-matrix metalloproteinase (MMP2) and MMP2 were semiquantified by scanning densitometry. Total MMP2 activity in DDR2^-/- ^expressed relative to that of DDR2^+/+^.

DDR2^-/- ^mice and DDR2^+/+ ^littermates were subjected to dermal incisions, and allowed to heal for up to 12 days. Whereas hematoxylin and eosin (H&E) staining of DDR2^-/- ^dermal scar sections 4.5 days post wounding were comparable to those of DDR2^+/+ ^scars [15], closure of DDR2^-/- ^wounds was significantly delayed (Figure 1a), with maximal differences between DDR2^-/- ^and DDR2^+/+ ^mice at day 4. The wounds of DDR2^-/- ^mice remained significantly larger than DDR2^+/+ ^mice for the following 7 days.

The rate of tissue healing depends largely on efficient recruitment of fibroblasts to the wound. Delayed DDR2^-/- ^mouse wound healing might be ascribed to delayed fibroblast recruitment to the granulation tissue. Western blot analysis of DDR2^-/- ^skin extracts 7 days post wounding revealed an average of 45% reduction in expression of αSMA, a marker of contractile wound myofibroblasts (Figure 1b). Using gel zymography, we previously detected a 60% reduction in MMP2 activity in unwounded DDR2^-/- ^mouse skin extracts [18]. Gelatin zymography analysis of skin extracts 7 days post wounding revealed two bands of gelatin degradation at 68 kDa and 62 kDa, representing latent and active MMP2, respectively (Figure 1c). MMP2 activity was reduced by an average of 75% in the DDR2^-/- ^wound extracts compared to DDR2^+/+ ^ones. These data are in accordance with our previous *in vitro *observations demonstrating reduced MMP2-dependent chemotactic migration of isolated αSMA-expressing DDR2^-/- ^skin fibroblasts [18].

**Figure 2:**
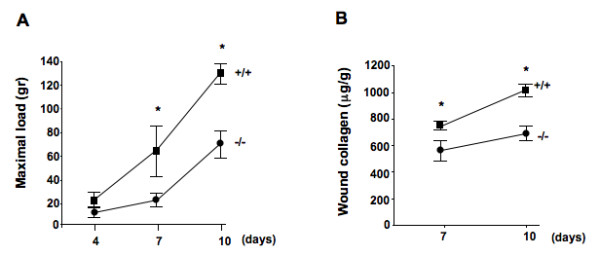
**Reduced tensile strength of discoidin domain receptor 2 (DDR2)^-/- ^wounds**. **(a) **Representative histogram of tensile mechanical testing in DDR2^-/- ^and DDR2^+/+ ^wounded skins (n = 6). Results are expressed as maximal load to failure (g). **(b) **Representative colorimetrical analysis of acid-soluble collagen amounts in skin lysates (n = 6) 7 days after wounding. Wounds were homogenized in lysis buffer and clarified by centrifugation. Total soluble collagen was measured colorimetrically at 540 nm using the Sircol Collagen Assay kit. *P < 0.001, significantly different compared with DDR2^+/+ ^group.

We next evaluated the tensile strength of skin wound closure. At 7 days after wounding, the maximal load before rupture in DDR2^-/- ^wounds was an average 65% less than in DDR2^+/+ ^wounds. The differences remained significant at day 10, where maximal load before rupture was 52% less in DDR2^-/- ^mice (Figure 2a).

Collagen is a key regulator of tissue tensile strength. Using a colorimetric assay, we observed that the collagen concentration in DDR2^-/- ^wounds was an average of 25% less than in DDR2^+/+ ^wounds 7 days after wounding, and an average of 40% less after 10 days (Figure 2b). These data correlate with our previous description of reduced collagen type I mRNA expression in cultured DDR2^-/- ^skin fibroblasts [18]. There were no significant differences in collagen content between unwounded DDR2^-/- ^and DDR2^+/+ ^mouse skin (data not shown).

**Figure 3:**
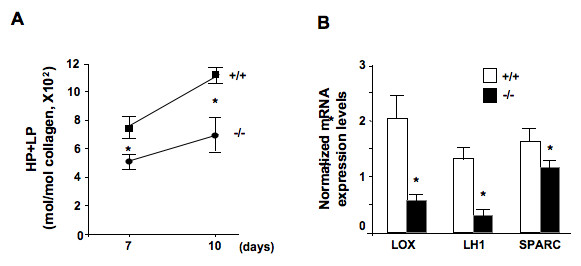
**Reduced gene expression for crosslinking enzymes in discoidin domain receptor 2 (DDR2)^-/- ^tissues**. **(a) **Representative histogram of trivalent lysylpyridinoline (LP) and hydroxylysylpyridinoline (HP) crosslinks. **(b) **Representative lysyl oxidase (LOX), lysyl hydroxylase1 (LH1) and 'secreted protein, acidic and rich in cysteine' (SPARC) mRNA expression levels in skin lysates 7 days after injury (n = 6). *P < 0.05, significantly different compared with the DDR2^+/+ ^group.

Collagen fibrils must be crosslinked for proper alignment and function. Reverse high-performance liquid chromatography (HPLC) analysis of pyridoline allowed us to measure trifunctional collagen crosslinks in the wounded skin extracts. At 7 days after incision, total pyridoline content (lysylpyridinoline + hydroxylysylpyridinoline) was 60% of that in the wild-type samples and levels of total pyridoline remained significantly lower 10 days after wounding (Figure 3a). Collagen crosslinking is mediated by LOX and LH. Collagen crosslink formation is initially catalyzed by LOX. There are two subsequent crosslinking pathways, an allysine and a hydroxyallysine route [24]. Pyridinolines, lysylpyridinolines (LPs) and hydroxylysylpyridinolines (HPs), are trifunctional crosslinks, derived from the hydroxyallysine crosslink route. Finally, LH catalyzes the conversion of lysine into a hydroxylysine in the collagen molecule. Further collagen fibril growth and deposition is mediated by SPARC [22]. Quantitative real-time reverse transcription polymerase chain reaction (RT-PCR) experiments demonstrated reduced mRNA expression of LOX, LH1 and SPARC in DDR2^-/- ^skin tissue, with an average reduction of mRNA expression of 75%, 81% and 45%, respectively, compared to wild-type tissue (Figure 3b).

While collagen type I is a major component of remodeling tissue after wounding, other collagenous and non-collagenous ECM molecules, their respective cell receptors and MMPs bound to ECM all participate in tissue wounding. Expression of several ECM molecules is modulated by DDR2 overexpression in NIH-3T3 cells [25] and altered in DDR2^-/- ^hepatic stellate cells (Olaso, unpublished results). Thus, we compared mRNA expression levels of these genes between cultured DDR2^+/+ ^and DDR2^-/- ^skin fibroblasts. As shown in Table 1, DDR2 deficiency in skin fibroblasts correlates with reduced mRNA levels of collagen type I, collagen type VIII, fibrillin 1, MMP2 and MMP10, while it increases collagen type VI, syndecan 1, and α2 integrin mRNA expression, as assessed by real-time PCR. Expression levels of fibronectin, α3 integrin, DDR1 and MMP13 mRNAs were not significantly different. These results are in agreement with the data obtained in NIH-3T3 cells overexpressing DDR2 and in DDR2^-/- ^hepatic stellate cells ([25]; Olaso, unpublished results).

**Table 1:** mRNA Levels of extracellular matrix (ECM) molecules in cultured discoidin domain receptor 2 (DDR2)^+/+ ^and DDR2^-/^^- ^skin fibroblasts

mRNA	**DDR2**^**+/+**^	**DDR2**^**-/-**^	Expression when DDR2 is absent
Col I (α 1)	2.54 ± 0.04	1.34 ± 0.11	Down

Col VI (α 2)	0.31 ± 0.07	0.45 ± 0.05	Up

Col VIII (α 1)	0.56 ± 0.02	0.34 ± 0.05	Down

Syndecan1	1.01 ± 0.06	0.06 ± 0.10	Down

Fibrillin 1	0.95 ± 0.01	0.78 ± 0.02	Down

Fibronectin	0.17 ± 0.01	0.13 ± 0.04	NS

α2	0.92 ± 0.03	1.52 ± 0.03	Up

α3	0.67 ± 0.07	0.61 ± 0.04	NS

DDR1	0.94 ± 0.20	0.91 ± 0.38	NS

MMP2	0.94 ± 0.01	0.31 ± 0.08	Down

MMP9	ND	ND	ND

MMP10	0.57 ± 0.09	0.24 ± 0.03	Down

MMP13	1.34 ± 0.10	1.17 ± 0.09	NS

Relative mRNA expression of ECM molecules regulated by DDR2 expression in DDR2^+/+ ^and DDR2^-/- ^skin fibroblasts. Gene expression was normalized to RPL13 mRNA expression. ND indicates mRNA was not detected. Results are expressed as means ± standard error of the mean (SEM) (n = 3). All *P *< 0.05 except for NS.

MMP = matrix metalloproteinase; NS = not significantly different.

**Figure 4:**
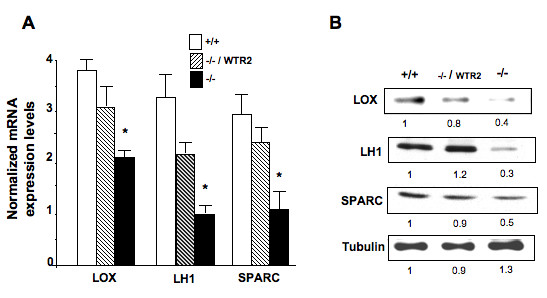
**Reconstitution of discoidin domain receptor 2 (DDR2) restores expression of crosslinking enzymes**. **(a) **Quantitative reverse-transcription real-time (RT)-PCR analysis of lysyl oxidase (LOX), lysyl hydroxylase1 (LH1) and 'secreted protein, acidic and rich in cysteine' (SPARC) mRNA levels in cultured skin fibroblasts. Results are expressed as relative to glyceraldehyde 3-phosphate dehydrogenase (GAPDH) mRNA expression. *P < 0.05, significantly different compared with DDR2^-/- ^skin fibroblasts retrovirally infected with full-length DDR2 (-/-/WTR2). **(b) **Representative western blot analysis of LOX, LH1 and SPARC protein levels in cultured skin fibroblasts. Tubulin expression was used as control for protein loading. Bands were quantified by scanning densitometry analysis and relativized to protein expression in DDR2^+/+ ^cells.

Next, we investigated if defective collagen crosslinking in DDR2^-/- ^skin correlates with reduced expression of collagen crosslinking molecules in DDR2^-/- ^fibroblasts. As shown in Figure 4, DDR2^-/- ^fibroblasts express significantly less LOX, LH1 and SPARC mRNAs (Figure 4a), and reduced LOX, LH1 and SPARC proteins (Figure 4b). We also analyzed DDR2-/- skin fibroblasts in which wild type DDR2 expression was reconstituted by retroviral infection (WTR2). We utilized WTR2 fibroblasts that expressed roughly similar levels of DDR2 wild-type cells (data not shown)[18]. LOX, LH1 and SPARC mRNA expression levels were restored in WTR2 cells and remained significantly higher than in the DDR2^-/- ^cells (Figure 4a, dashed columns). As shown in Figure 4b, LOX, LH1 and SPARC protein expression in WTR2 cells was similar to that of DDR2^+/+ ^cells. DDR2^-/- ^skin fibroblasts retrovirally infected with control vector (internal ribosomal entry site and green fluorescent protein (GFP) cDNA) behaved as non-infected cells (data not shown).

**Figure 5:**
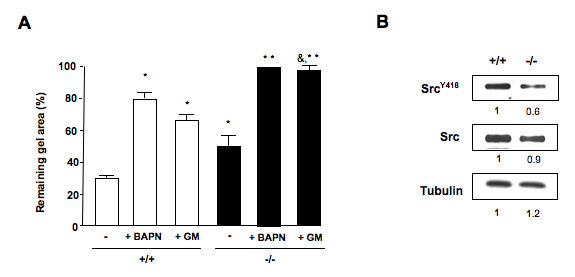
**Reduced contractility of discoidin domain receptor 2 (DDR2)^-/- ^fibroblasts**. **(a) **Representative histogram of collagen contraction assay. Collagen gels were seeded with 1 × 10^5 ^cells/mL fibroblast derived from DDR2^+/+ ^or DDR2^-/- ^mice or with DDR2^-/- ^skin fibroblasts retrovirally infected with wild-type DDR2 (-/-/WTR2). Cells were maintained for 2 days culture under basal conditions (control) or under keratinocyte supernatants with or without (untreated) 3 μM β-aminoproprionitrile (BAPN; a lysyl oxidase inhibitor) or 1 μM *N*-[(2R)-2-(hydroxamido carbonylmethyl)-4-methylpentanoyl]-l-tryptophan methylamide (GM6001; a matrix metalloproteinase (MMP) inhibitor). Then, collagen was removed from the well sides and soluble type I collagen (30 μg/ml) was added. Cells were allowed to contract the lattices for 72 h. Digital images were taken and the gel area was calculated. *P < 0.01, significantly different compared with untreated DDR2^+/+^; **P < 0.01, significantly different compared with untreated DDR2^-/- ^cells. ^&^*P *< 0.05, significantly different compared with GM-treated DDR2^+/+ ^cells. Control DDR2^+/+ ^and DDR2^-/- ^cells retained 85% and 95%, respectively, of initial gel area. **(b) **Representative western blot analysis for Src^Y418 ^and total Src expression by skin fibroblasts after gel contraction. Tubulin expression is utilized as loading control. Bands were quantified by scanning densitometry analysis.

Wound closure reflects contraction of the newly formed matrix by myofibroblasts. To assess the importance of DDR2 in cell contraction, skin fibroblasts were assayed for their capacity to reduce a collagen gel in response to a contractile stimulus (keratinocyte culture supernatants) and in the presence of DDR2 ligand (soluble collagen type I) (Figure 5a). DDR2^-/- ^skin fibroblasts activated by keratinocyte supernatants contracted collagen gels 40% less than DDR2^+/+ ^fibroblasts.

Defective matrix metalloproteinase expression is associated with *in vitro *fibroblast contraction [26] and *in vivo *impaired wound contraction in murine wound models [27,28]. Using the specific MMP inhibitor *N*-[(2R)-2-(hydroxamido carbonylmethyl)-4-methylpentanoyl]-l-tryptophan methylamide (GM6001), we previously reported that DDR2-dependent migration of skin fibroblast is also MMP dependent [18]. Herein, pretreatment of DDR2^+/+ ^and DDR2^-/- ^skin fibroblasts with 1 μM GM6001 completely blocked DDR2^-/- ^skin fibroblast contraction, but retained DDR2^+/+ ^skin's ability to contract 30% of the initial collagen gel area.

LOX activity also promotes fibroblast contraction [29]. Interestingly, under experimental conditions, pretreatment with the LOX inhibitor *N*-bromoacetylpyridoxamine (BAPM) allowed DDR2^+/+ ^skin fibroblasts to contract 20% of the initial collagen gel area, DDR2^-/- ^skin fibroblast contraction was completely blocked, indicating reduced LOX activity dependent fibroblast contraction in the DDR2^-/- ^cells. Under the assayed conditions, neither GM6001 nor β-aminoproprionitrile (BAPN) affected fibroblast survival, as demonstrated by trypan blue exclusion test (data not shown).

Fibroblast contraction is mediated by focal adhesion kinase (FAK)/Src signaling [30]. Skin fibroblasts were recovered from contracted collagen gels and analyzed by western blot (Figure 5b). While there were no significant differences in total Src, DDR2^-/- ^fibroblasts expressed an average of 30% less phosphorylated Src^Y418 ^than DDR2^+/+ ^ones, which may correlate with their reduced contraction ability.

## Discussion

Fibroblasts are major effectors of wound repair and regenerative healing. They coordinate the deposition and further organization of the collagenous extracellular matrix in the wound bed, and thereby determine the final outcome of the scarring process. We have previously shown that fibroblasts from DDR2 knockout mice are defective in key aspects of cell proliferation and ECM remodeling [18]. In this work, we further explore DDR2^-/- ^skin fibroblasts in the context of experimental cutaneous wound healing. Furthermore, we identify new molecules tightly related to ECM assembly whose expression is blunted in fibroblasts from wounded DDR2^-/- ^skin. Because reconstitution of DDR2 expression by retroviral infection of DDR2^-/- ^skin fibroblasts resulted in almost full recovery of their mRNA levels to that of DDR2^+/+ ^cells, we conclude that the observed effects were attributable to DDR2.

While there is considerable information about DDR1 signaling [31,32], DDR2 signaling is poorly characterized [33]. We have previously shown that Src kinase and Shc recruitment are key signaling intermediates in DDR2 signal transduction following collagen type I induction of tyrosine 740 phosphorylation [34]. Src phosphorylation at tyrosine 418 is required for maximal DDR2 phosphorylation [35], resulting in increased MMP2 promoter activity. Furthermore, DDR2 is involved in the regulation of FAK levels in vascular smooth muscle cells adherent to type I collagen matrices [36].

DDR2 deficiency in skin fibroblasts correlates with reduced mRNA levels of collagen type I and MMP2 [18]. In this work, we compared mRNA and protein expression between DDR2^+/+ ^and DDR2^-/- ^skin fibroblasts and describe a cluster of ECM-related genes regulated by DDR2 signaling, including collagen type VI, collagen type VIII, syndecan 1, SPARC, LOX, LH1 and MMP10.

DDR2^-/- ^skin wounds show delayed closure and impaired recovery of tensile strength, which may be ascribed to a defective ability of DDR2^-/- ^fibroblasts to synthesize the ECM in the newly formed healing tissue. The observed reduced content of fibrillar collagen is the result of downregulation of collagen expression, alteration in matrix remodeling through variation in MMP composition, and reduced collagen crosslinking by LOX, LH1 and SPARC. Interestingly, the extracellular domain (ECD) of DDR2, when used as a purified, soluble protein, inhibits collagen fibrillogenesis *in vitro *[37].

Our work demonstrates the delayed closure of DDR2^-/- ^dermal wounds, and impaired contraction on collagen type I gels by DDR2^-/- ^skin fibroblasts. Fibroblast contraction is a key step in dermal wound healing, where mechanical signals derived from the extracellular matrix induce mechanoreceptor signaling and activation of the contractile machinery. While integrins were the first mechanoreceptors described, increasing evidence demonstrates a role for receptor tyrosine kinases (RTKs) in cell motility that may relay on interactions between integrins and RTK signaling pathways, and activation of the FAK/Src signaling pathway [38-40]. While no evidence exists for integrin-DDR interaction at the plasma membrane, convergence of their downstream signaling may occur. In this regard, Src phosphorylation is a downstream event following both integrin α2β1 and DDR2 mediated cell contraction, as DDR2^-/- ^fibroblasts contain less phosphorylated Src^Y418 ^than wild-type cells. Interestingly, collagen stimulation of DDR1 mediates migration of smooth muscle cells through Src [41].

As described in retinal pigment epithelium (RPE) cells [31], skin fibroblasts require the FAK/Src complex for collagen gel contraction. DDR2 is involved in the regulation of FAK levels [36] but its activation does not modify cellular adhesion [18]. Furthermore, only anti-integrin α2 antibody-treated RPE cells showed a statistically significant, albeit modest decrease in adhesion as compared to wild-type cells.

DDR2^-/- ^skin fibroblasts express higher mRNA levels of other receptors for fibrillar collagen such as integrin α2. However, mRNA expression of DDR1, the other member of the DDR family is not significantly modified in skin fibroblasts. Interestingly, α2 and DDR1 expression are increased in DDR2^-/- ^stellate cells from carbon-tetrachloride intoxicated livers (E. Olaso, unpublished results). Thus, upregulation of α2 and/or DDR1 may compensate for DDR2 deficiency during cell adhesion.

The motogenic phenotype of skin fibroblasts is regulated by MMP-mediated remodeling of its associated ECM, and the MMP inhibitor GM6001, specifically impairs wound contraction [26]. GM6001 also inhibits DDR2-mediated invasion into basement membrane matrix by hepatic stellate cells [10]. In this work we demonstrate impaired contraction of collagen gels in skin fibroblasts treated with GM6001.

DDR2^-/- ^skin fibroblasts express reduced levels of LOX and deficient cell contraction, and blockade of LOX activity by BAPN results in reduced skin fibroblast contraction. A growing body of evidence shows that LOX plays a key role in intracellular signaling underlying cell motogenicity. LOX activity promotes focal adhesions through FAK/Src signaling [13] and crosslinked collagen induces integrin clustering that enhances PI3K signaling, and induces invasion of premalignant epithelium. Ongoing studies on the interaction of LOX and DDR2 signaling in the tumor microenvironment may further explain the relationship between collagen dysregulation and cancer invasion and metastasis.

## Conclusions

Based on our findings to date we propose multiple interrelated features are altered by DDR2 signaling in skin fibroblasts, which may affect skin wound healing and tensile strength: (1) chemotactic migration to the wounded area and proliferation, (2) synthesis and remodeling of the wound matrix, (3) three-dimensional organization of the wound matrix by collagen cross linking and collagen fibrillogenesis, and (4) fibroblast contraction of the healing wound.

## Methods

### Cell isolation and culture

The DDR2^-/- ^mice and derived skin fibroblasts have been described previously [15,18]. In brief, skin fibroblasts from knockout or wild-type littermate 6-8-week-old male mice were isolated from ear lobules and later transfected with 10 μg of the SV40 large T antigen cDNA. Skin fibroblasts from DDR2^-/- ^mice were infected with a retrovirus containing a bicistronic construct encoding either the full length DDR2 (WTR2) Following these sequences was a cassette encoding enhanced (e)GFP under the control of an internal ribosomal entry site. Cells expressing high levels of eGFP were recovered by fluorescence-activated cell sorting (FACS) (Dako MoFlo, Cytomation), as previously reported [18,34]. The same results were obtained using untransfected DDR2^-/- ^cells, or DDR2^-/- ^cells infected with a retroviral vector expressing only the internal ribosomal entry site and eGFP cDNA (data not shown).

Keratinocytes were obtained from the American Tissue Culture Collection http://www.atcc.org. Then, 6 h after calf serum removal, basal media was added to 30% confluent cell cultures and collected after overnight conditioning. It was then centrifuged to eliminate cellular debris and stored at -80°C.

### Experimental skin injury

Male, 6-8-week-old mice were used. The dorsal skin was shaved and cleaned with Betadine (Purdue Products L.P., Stamford, CT) under anesthesia, and the skin was incised up to the level of the subcutaneous adipose tissue with a 5-mm Acu-Punch biopsy tool (Acuderm; Fort Lauderdale, FL, USA). A sterile dressing was placed around the trunk in order to protect against infection. On days 4, 7 and 10 after incision the remaining wound was measured using a caliper. Then, the entire dorsal skin was excised and processed for further analysis. All animal studies were performed with approval of the institutional animal welfare committee.

### Tensile strength measurement

Skin samples were excised from DDR2^-/- ^and DDR2^+/+ ^mice. Strips with a cross sectional area of 2 mm × 6 mm were removed from the mid portion of the wound and subjected to tensile mechanical testing on an Instron Series 3340 (Instron Corporation, Canton, MA, USA). Tensile testing was performed at a strain rate of 417 μm/s using a 51 kg load cell.

### Collagen (Sircol) assay

Total acid-soluble collagen (types I-V) in wound tissue was measured colorimetrically using a Sircol Collagen Assay kit (Newtown Abbey, UK) according to the manufacturer's instructions. Briefly, wound tissue sample was homogenized in lysis buffer (100 mM potassium phosphate, 0.1% Triton X-100, 2 mM dithiothreitol, 100 μg/mL phenylmethylsulfonyl fluoride, pH 7.8) and tissue debris was removed by centrifugation. Sircol dye reagent was added to tissue extracts, stirred for 30 min at room temperature and centrifuged at 10,000 *g *for 15 min. Absorbance of the bound dye was measured at 540 nm in a spectrophotometer. The amount of collagen protein in skin samples was adjusted to the amount of total protein using the BCA Protein Assay kit (Pierce, Rockford, IL, USA). Collagen concentrations were expressed as μg collagen per gram of total protein.

### Gelatin zymography

Wound tissues were extracted and immediately frozen at -20°C to prevent autoactivation of MMP2. Aliquots from same total protein amounts were later analyzed by gelatin zymography on 8% polyacrylamide gels as previously described [42]. Results were quantified using the Quantity One software package (Bio-Rad, Hercules, CA, USA).

### Quantitative RT-PCR

First-strand complementary DNA (cDNA) was generated from 5 μg of total RNA with the Moloney murine leukemia virus reverse transcriptase (Gibco Life Technologies, Gaithersburg, ML, USA). Quantitative real-time PCR reactions were performed in triplicate with the Syber Green PCR Core Reagent kit and the Gene Amp 5700 sequence detection system using commercially available primer sets (all from Applied Biosystems, Foster City, CA, USA).

### Measurement of pyridinoline crosslinking

Lyophilized tissues were hydrolyzed in 6 M HCl at 100°C for 30 h and dried, dissolved in an internal standard solution (10 uM pyridoxine and 2.40 mM homoarginine in water) and further diluted 1:4 in 0.5% heptafluorobutyric acid in 10% acetonitrile. HP and LP were measured in acid hydrolysates of unreduced samples by reversed-phase high-performance liquid chromatography. The amount of crosslinking was calculated based on an internal standard containing pyridoxine, HP, and LP.

### Cell lysis, immunoprecipitation, and immunoblotting

Skin fibroblasts were lysed in radioimmunoprecipitation assay (RIPA) buffer (50 mM Tris (pH 8), 150 mM NaCl, 1% Triton X-100, 0.5% deoxycholate acid, and 0.1% SDS) containing protease inhibitors (Roche Farma, Barcelona, Spain), 2 mM sodium orthovanadate, and 10 mM sodium fluoride. Insoluble material was removed by centrifugation at 4°C. Total cell protein in lysates from serum-starved cells was determined by a Bradford assay (Bio-Rad). The conditioned medium was harvested and concentrated 20-fold (Centriplus concentrators, 10 kDa MW cut-off; Millipore, Bedford, Massachusetts, USA). Then, 50 μg of cell lysate and 25 μg of concentrate media were separated through SDS-PAGE (10% acrylamide gel) and blotted onto a nitrocellulose membrane (Bio-Rad). Monoclonal antibodies were used to detect SPARC, tubulin (both 1:1,000; Upstate, Billerica, MA, USA) and αSMA (1:1,000, Sigma) (Sigma Aldrich, St Louis, Mo), following by anti-mouse IgG (1:5,000, Upstate). LOX was detected in the cell lysates and LH1 was detected in the concentrate media. Both were detected using polyclonal antibodies against internal region of the proteins (1:100) followed by the appropriate secondary anti-goat or anti-rabbit IgG (1:5,000), all from Santa Cruz Biotechnology (Santa Cruz, CA, USA). The bands were visualized using the Super Signal Femto Substrate kit (Pierce) and further semiquantified by the Quantity One software (Bio-Rad).

Src was detected after immunoprecipitation with protein A-agarose beads (Sigma). The immunoprecipitated proteins were washed at 4°C in the lysis buffer prior to direct analysis by SDS-PAGE (8% acrylamide gel) and blotted onto a nitrocellulose membrane (Bio-Rad). The blots were incubated with anti-Src-pTyr418 (1:1,000; Biosource) (Invitrogen Biosource, Carlsbad, California) and anti-Src (1:250; Upstate, Billerica, MA, USA)). Bound primary antibody was visualized by chemiluminescent horseradish peroxidase substrate (Pierce) with appropriate horseradish peroxidase-conjugated antibody (1:5,000; Upstate). Membranes were stripped of bound antibodies by incubation in buffer (62.5 mM Tris-HCl, pH 6.8, 2% SDS, 100 mM 2-mercaptoethanol) at 55°C for 30 min prior to reprobing. The bands were visualized using the Super Signal Femto Substrate kit (Pierce) and further semiquantified by the Quantity One software (Bio-Rad).

### Collagen contraction assays

Contraction of collagen gels on collagen lattices was performed as previously described [43] with some modifications. In brief, culture vessels were preincubated with 0.5% BSA and washed twice. A total of 500 μl of ice-cold collagen solution (16 ml of Vitrogen 100 purified collagen type I (Collagen Corp., Palo Alto, CA, USA), 2 ml 10 × Dulbecco's modified Eagle medium (DMEM) and 2 ml of 0.2 M 4-(2-hydroxyethyl)-1-piperazineethanesulfonic acid (HEPES)) was added to each bovine serum albumin (BSA)-treated well and incubated for 1 h to allow gelation. Then, 1 × 10^5 ^fibroblasts in 500 μl DMEM were layered on top of the wells. A total of 1 ml of keratinocyte culture supernatants or serum-free DMEM was added to each well, with or without 3 μM BAPN (a lysyl oxidase inhibitor) or 1 μM GM6001 (an MMP inhibitor), and cells were cultured for 2 days. Collagen was removed from the well sides with a sterile scalpel, soluble collagen was added to the cultures (30 μg/ml) and cells were allowed to contract the lattices for 72 h. Under the study conditions, cell viability was not modified by *N*-bromoacetylpyridoxamine (β-AMPN) or GM6001, as demonstrated by the trypan blue exclusion and 3-(4,5-dimethylthiazol-2-yl)-2,5-diphenyltetrazolium bromide (MTT) assay. Digital images were captured and the gel area was calculated. In some experiments, fibroblasts were recovered from the collagen gel after treatment with 4 mM ethylenediaminetetraacetic acid (EDTA) solution in phosphate-buffered saline (PBS).

### Statistical analysis

Statistical results refer to mean ± SD. Statistical analysis was performed using SPPS (SPSS, Chicago, IL, USA). Individual comparisons were made with the Student's two-tailed, unpaired *t *test (Statview, Abacus Concepts, Berkeley, CA, USA). The criterion for significance was *P *< 0.05 for all comparisons. *In vivo *experiments were performed with three mice per group and repeated at least twice. *In vitro *experiments were performed with three mice per group at least in duplicate and repeated at least twice. The data presented in the Results section were obtained as the average numbers of the two or three experiments.

## Competing interests

The authors declare that they have no competing interests. LHW and HSL are employees of Regeneron Pharmaceuticals, Ltd.

## Authors' contributions

EO carried out *in vivo *and *in vitro *experiments, conceived the study and drafted the manuscript. HSL and LHW carried out skin wounding assays and provided the transgenic mice and skin fibroblasts. SLF contributed to the study design and data interpretation and participated in the drafting of the manuscript. All authors read and approved the final manuscript.
